# Transcriptomics and Physiological Analysis Reveal Response Strategies of *Sanghuangporus baumii* to Aluminum Exposure

**DOI:** 10.3390/jof12080586

**Published:** 2026-08-07

**Authors:** Xinyu Tong, Anxin Wang, Zengcai Liu, Li Zou

**Affiliations:** College of Forestry, Northeast Forestry University, Harbin 150040, China; 18846783859@163.com (X.T.); 17661291539@163.com (A.W.)

**Keywords:** *Sanghuangporus baumii*, aluminum, antioxidant enzyme, glutathione metabolism, soluble sugar, triterpenoids

## Abstract

*Sanghuangporus baumii*, a medicinal macrofungus, remains unexplored in its response to aluminum stress, despite the widespread environmental relevance of this metal. This study investigated the dose-dependent effects of Al^3+^ on mycelial growth and metabolic regulation. Exposure to 1 mM Al^3+^ moderately stimulated growth (1.11-fold of control) and induced mild oxidative stress, which activated an effective antioxidant response—including increased SOD, CAT, and POD activities and elevated reduced glutathione content—thereby maintaining redox balance and increasing soluble sugar content. In contrast, 10 mM Al^3+^ led to pronounced intracellular Al^3+^ accumulation, severe growth inhibition, and marked oxidative damage, accompanied by impairment of the antioxidant system, yet a marked increase in total triterpenoid content (1.72-fold). Transcriptomic analysis identified 642 and 3019 differentially expressed genes (DEGs) in the 1 and 10 mM Al^3+^ treatments, respectively. KEGG enrichment analysis revealed concentration-dependent alterations in pathways related to peroxisome function, glutathione metabolism, starch and sucrose metabolism, and terpenoid backbone biosynthesis. Collectively, these findings suggest that Al^3+^ modulates *S. baumii* growth and metabolism in a dose-dependent manner, with redox remodeling as a potential mechanism, offering novel insights into fungal metal adaptation and the targeted modulation of medicinal metabolite production.

## 1. Introduction

*Sanghuangporus baumii* is a valuable medicinal macrofungus with a long history in traditional Chinese medicine. It is widely recognized for its antitumor, hypoglycemic, and immunomodulatory activities, and its market demand is growing rapidly [1,2,3]. However, inconsistent product quality remains a major bottleneck restricting its industrial development. Accumulating evidence suggests that environmental factors profoundly regulate the growth and development of macrofungi [4,5,6]. In particular, metal ions not only affect mycelial growth but also play a crucial role in the accumulation of bioactive metabolites in macrofungi [7,8].

Aluminum (Al) is the most abundant metallic element in the Earth’s crust, and its trivalent ionic form (Al^3+^) is a ubiquitous environmental factor that *S. baumii* may encounter throughout its life cycle, from mycelial growth to fruiting body development. In plants, low concentrations of Al^3+^ promote growth, whereas high concentrations induce oxidative stress and inhibit development [9,10,11]. A previous study reported concentration-dependent effects of Al^3+^ on fungal growth, with relatively low concentrations promoting mycelial growth in *Aspergillus oryzae* and higher concentrations inhibiting vegetative growth and reproductive development [12]. However, the physiological and molecular mechanisms underlying these responses remain poorly understood. Mechanistic evidence is currently derived mainly from the model yeast *Saccharomyces cerevisiae*, in which Al^3+^ exposure has been associated with ROS accumulation, lipid peroxidation, membrane damage, and growth inhibition [13]. In macrofungi, previous studies have mainly focused on the ability of ectomycorrhizal fungi to alleviate aluminum toxicity in host plants [14,15]. By contrast, the intrinsic redox and metabolic responses of macrofungi, particularly medicinal species such as *S. baumii*, remain largely unexplored.

In this study, we investigated whether aluminum exposure alters redox homeostasis in *S. baumii* and whether such alterations are associated with mycelial growth and secondary metabolite accumulation. We hypothesized that aluminum-associated redox remodeling is linked to the balance between biomass production and triterpenoid accumulation. To test this hypothesis, we first examined the effects of Al^3+^ on mycelial growth and identified concentrations that promoted or inhibited growth. We then performed transcriptomic profiling and systematic physiological and biochemical measurements, including redox status, antioxidant enzyme activities, glutathione content, soluble sugar levels, and total triterpenoid content, on mycelia exposed to these two contrasting conditions. By integrating transcriptomic and physiological data, we aimed to elucidate the key metabolic pathways and regulatory mechanisms underlying Al^3+^ responses in *S. baumii*. These findings provide a foundation for understanding how macrofungi adapt to metal stress and shed light on metal-induced metabolic regulation of medicinal macrofungi.

## 2. Materials and Methods

### 2.1. Al^3+^ Treatment and Growth Determination

A single strain of *Sanghuangporus baumii* was used in this study. The strain was isolated from a wild fruiting body collected from the living trunk of *Syringa reticulata* in Liangshui Nature Reserve, Yichun, Heilongjiang, China. Species identification was based on morphological characteristics and internal transcribed spacer (ITS) sequence analysis. The ITS sequence was deposited in GenBank under accession number MT804594. The culture was maintained on potato dextrose agar (PDA) slants at 4 °C in the Laboratory of Forest Protection, Northeast Forestry University, and was reactivated on fresh PDA at 25 °C in darkness before use.

To evaluate the effects of Al^3+^ on the growth of *S. baumii*, two culture systems were used for different experimental purposes. Solid-medium assays were used to evaluate colony growth during continuous Al^3+^ exposure from the time of inoculation. Liquid cultures were used to assess biomass responses and to obtain sufficient and relatively uniform mycelial material for subsequent physiological and transcriptomic analyses after the establishment of mycelial growth. The culture media were supplemented with a filter-sterilized AlCl_3_ stock solution to achieve nominal concentrations of 0, 0.01, 0.1, 1, 2, 5, and 10 mM.

For the solid-medium assay, each PDA plate was centrally inoculated with one 5-mm-diameter agar plug excised from the actively growing margin of a mycelial colony. The plates contained the designated nominal Al^3+^ concentration and were incubated at 25 °C in darkness for 8 d. Mycelial colony morphology was documented and the growth rate was calculated as (D − d)/2n (cm/d), where D is the colony diameter, d is the agar plug diameter, and n is the number of days of incubation.

To prepare the liquid seed culture, fifteen 5-mm-diameter mycelial agar plugs were inoculated into 200 mL of potato dextrose broth (PDB) in a 500-mL Erlenmeyer flask and cultured for 10 d. The resulting culture was homogenized using a Waring homogenizer (Waring, Torrington, CT, USA). The homogenized seed culture was then inoculated into fresh PDB at 4% (*v*/*v*) and pre-cultured for 8 d before the addition of AlCl_3_. The initial pH of the medium, measured before inoculation, was approximately 6.0 and was not adjusted. Medium pH was not monitored during the subsequent exposure period. After 48 h of exposure, the mycelia were harvested for dry biomass determination and subsequent physiological, biochemical, and transcriptomic analyses. The 48 h exposure period was selected based on preliminary time-course experiments (2–72 h) with other metal ions in *S. baumii*, in which 48 h yielded measurable responses and sufficient biomass for subsequent analyses. The same exposure duration was also used in our previous study of metal-induced metabolic responses in this species [16]. All liquid cultures were incubated at 25 °C and 180 rpm in darkness.

The intracellular accumulation of Al^3+^ in *S. baumii* mycelia was quantified by inductively coupled plasma mass spectrometry (iCAP TQ, Thermo Fisher Scientific, Waltham, MA, USA) following a standard protocol [17].

### 2.2. Transcriptome Sequencing of Mycelial Samples After Al^3+^ Treatment

*S. baumii* mycelial samples from the growth-promoting and growth-inhibiting Al^3+^ treatments, along with the untreated control, were selected for transcriptome analysis. Total RNA was extracted from these samples and subjected to sequencing on an Illumina HiSeq 2500 platform by PANOMIX Biomedical Tech Co., Ltd. (Suzhou, China). Briefly, mRNA was enriched from total RNA using oligo(dT) magnetic beads and subsequently fragmented into 200–300 bp segments. First-strand and second-strand cDNAs were synthesized, followed by library construction. After quality assessment, the libraries were sequenced to generate paired-end reads. A total of nine libraries were constructed, corresponding to three experimental conditions, each with three biological replicates. Raw reads were quality-filtered to obtain clean reads, which were subsequently assembled de novo using Trinity to generate unigenes. Transcript abundance was estimated as fragments per kilobase of transcript per million mapped reads (FPKM). Differential expression analysis was performed using DESeq2 on the gene-level raw-count matrix; genes with |log_2_FC| > 1 and Benjamini–Hochberg-adjusted *p*-value < 0.05 were classified as DEGs and functionally annotated against NR (NCBI non-redundant protein sequences), GO (Gene Ontology), KEGG (Kyoto Encyclopedia of Genes and Genomes), eggNOG (evolutionary genealogy of genes: Non-supervised Orthologous Groups), Swiss-Prot, and Pfam databases. To validate the transcriptomic results, the expression patterns of nine selected DEGs were confirmed using quantitative real-time polymerase chain reaction (qRT-PCR). The gene-specific primers used in this assay are listed in Table S1.

### 2.3. Detection of Oxidative Stress Markers

The contents of malondialdehyde (MDA) and hydrogen peroxide (H_2_O_2_) in mycelia were quantified using commercial assay kits (MDA-2-Y and H_2_O_2_-2-Y; Suzhou Comin Biotechnology Co., Ltd., Suzhou, China), according to the manufacturer’s protocols. To visualize the accumulation of superoxide anion (O_2_^−^), mycelial samples subjected to Al^3+^ treatment were collected, rinsed thoroughly with phosphate-buffered saline (PBS) to remove residual culture medium, and subsequently incubated in PBS containing 0.5 mg/mL nitroblue tetrazolium (NBT). The staining was performed in the dark at 25 °C for 1 h. After incubation, the mycelia were washed again with PBS to remove excess dye. The intensity of the dark blue formazan precipitate, indicative of O_2_^−^ production, was then documented by imaging for qualitative assessment.

### 2.4. Evaluation of the Antioxidant System

Mycelial samples were washed with sterile water, collected by centrifugation at 5000× *g* for 10 min, and immediately stored at −80 °C. The frozen mycelia were ground to a fine powder under liquid nitrogen. For each assay, 0.1 g of mycelial powder was mixed with 1 mL of the ice-cold extraction buffer supplied with the corresponding assay kit and homogenized on ice according to the manufacturer’s instructions. The extraction buffer composition was proprietary and was not disclosed by the manufacturer. The resulting homogenate was centrifuged at 12,000× *g* for 10 min at 4 °C, and the supernatant was carefully collected. The activities of key antioxidant enzymes, including superoxide dismutase (SOD), catalase (CAT), and peroxidase (POD), and the content of reduced glutathione (GSH) and its oxidized form (GSSG), were measured using commercial kits (SOD-2-W, CAT-2-W, POD-2-Y, GSH-2-W, and GSSG-2-W; Suzhou Comin Biotechnology Co., Ltd., China) according to the manufacturers’ protocols. The GSH/GSSG ratio was calculated to evaluate the cellular redox status.

### 2.5. Quantification of Soluble Sugar Content and Total Triterpenoid Content

The soluble sugar content was quantified with a specific assay kit (KT-2-Y; Suzhou Comin Biotechnology Co., Ltd., China). Total triterpenoid content was determined following an established colorimetric procedure as described by Liu et al. [16].

### 2.6. Data Analysis

Each treatment comprised three independent biological cultures. For each biological replicate, each physiological measurement was performed in triplicate. Technical measurements were averaged within each biological replicate, and the biological replicate was treated as the experimental unit. Data are presented as mean ± SD. Different lowercase letters indicate statistically significant differences at *p* < 0.05. All statistical analyses were performed using IBM SPSS Statistics version 25.0.

## 3. Results

### 3.1. Dose-Dependent Effects of Al^3+^ on Mycelial Growth and Intracellular Accumulation

**Figure 1:**
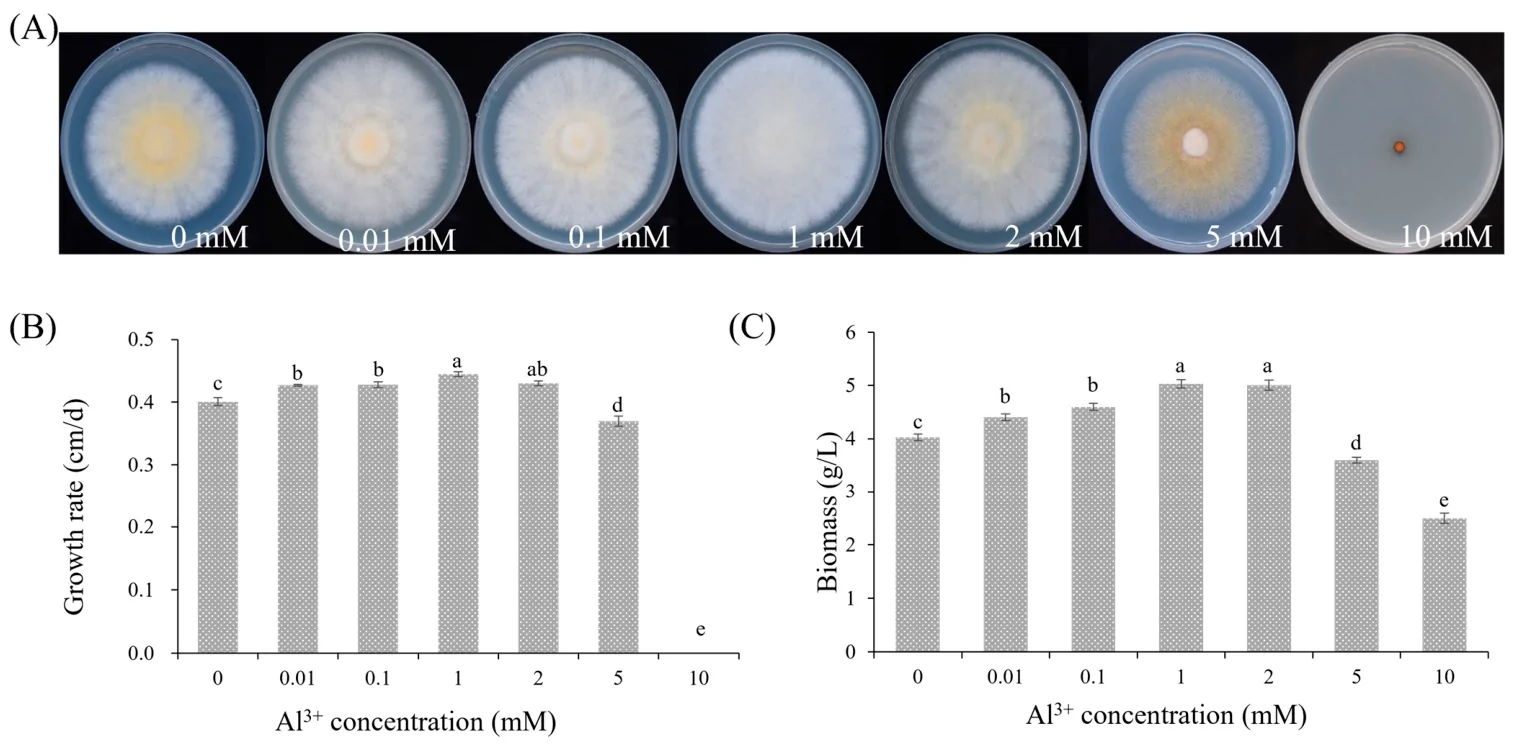
Effects of Al^3+^ treatment on *S. baumii* growth. (**A**) Colony morphology. (**B**) Mycelial growth rate. (**C**) Mycelial biomass. Letters indicate a significant difference (*p* < 0.05).

To assess the response of *S. baumii* to Al^3+^, mycelial growth rate and biomass accumulation were examined under a gradient of exogenous Al^3+^ concentrations. As shown in Figure 1, Al^3+^ exposure produced a clear biphasic effect on mycelial development. Colony diameter increased as the Al^3+^ concentration rose from 0 to 2 mM, with a maximum growth rate of 0.450 ± 0.004 cm/d at 1 mM, which was 1.11-fold that of the control (0.40 ± 0.01 cm/d). Concurrently, the yellow pigmentation at the colony center gradually faded within the 0–1 mM range, reaching its lightest intensity at 1 mM, and then slightly intensified at 2 mM, though remaining lighter than the control. In contrast, further increasing the Al^3+^ concentration to 5 or 10 mM progressively reduced the colony diameter, with almost no mycelial growth observed at 10 mM. This growth inhibition was accompanied by a marked intensification of yellow pigmentation, suggesting a change in metabolic status. Although the solid- and liquid-culture systems were not quantitatively comparable, the liquid-culture biomass data exhibited the same directional response as the solid-medium growth data at the key treatment concentrations (Figure 1C). Biomass increased from 4.03 ± 0.06 g/L in the control to 5.04 ± 0.08 g/L under 1 mM treatment, but decreased to 2.49 ± 0.10 g/L under 10 mM treatment. Given this qualitative agreement and the suitability of liquid culture for obtaining sufficient mycelial material, liquid-cultured samples were used for the subsequent biochemical and transcriptomic analyses.

To further examine the relationship between growth responses and Al^3+^ uptake, intracellular Al^3+^ contents were measured under the growth-promoting (1 mM) and growth-inhibiting (10 mM) conditions (Figure S1). Intracellular Al^3+^ accumulation increased with increasing exogenous Al^3+^ concentration. The intracellular Al^3+^ content was 3.53 ± 1.66 μg/g in the control, 31.16 ± 2.02 μg/g in the 1 mM treatment, and 475.22 ± 42.73 μg/g in the 10 mM treatment, representing 8.83- and 134.63-fold that of the control, respectively. Collectively, these results indicate that exogenous Al^3+^ regulates mycelial growth in a manner correlated with its intracellular accumulation.

### 3.2. Effects of Al^3+^ on Metabolic Pathways and DEGs

To elucidate the molecular mechanisms underlying the response of *S. baumii* to Al^3+^, transcriptome sequencing was performed using mycelia from three conditions, including the untreated control (Al_0_ group), the growth-promoting 1 mM Al^3+^ treatment (Al_1_ group), and the growth-inhibiting 10 mM Al^3+^ treatment (Al_10_ group). De novo assembly generated 8621 unigenes with a total length of 23.43 Mb, an N50 of 4025 bp, and a mean length of 2718 bp (Table S2; Figure S2). Sequencing of the nine libraries yielded 393,184,870 clean reads, with an average Q30 value of 96.13% (Table S3). Of the 8621 unigenes, 1834 (21.27%) were annotated in all six databases. In total, 7156 unigenes (83.01%) were annotated against NR, 3087 (35.81%) against GO, 3032 (35.17%) against KEGG, 5638 (65.40%) against eggNOG, 4639 (53.81%) against Swiss-Prot, and 4339 (50.33%) against Pfam.

**Figure 2:**
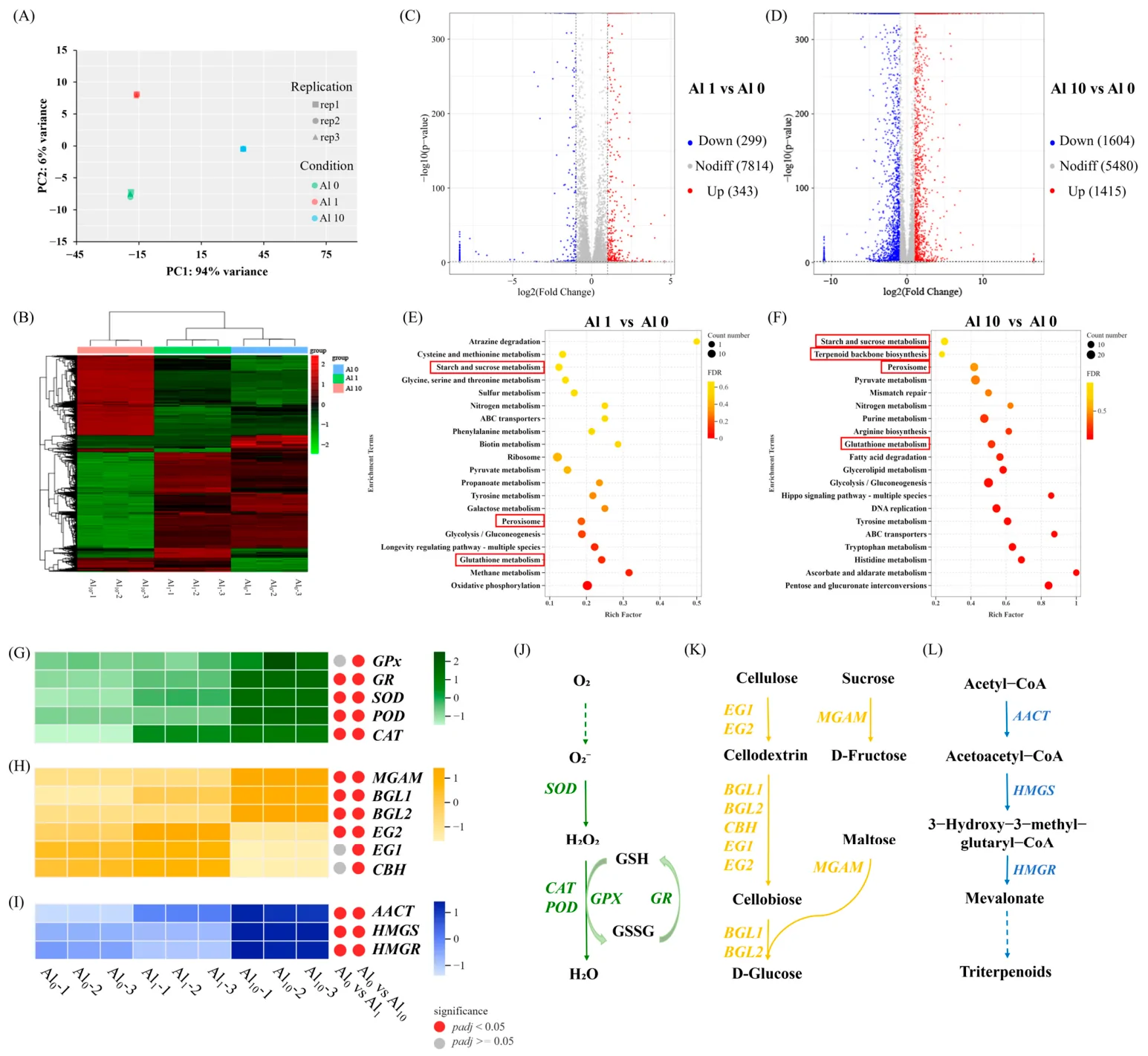
RNA-seq data analysis of *S. baumii* under Al^3+^ treatment. (**A**) PCA of Al_0_, Al_1_, and Al_10_ groups. (**B**) Heatmap of sample clustering among the three groups. (**C**,**D**) Volcano plots of DEGs. The dotted horizontal line indicates the threshold of -log10(*p*-value) = 0 (i.e., *p*-value = 1), serving as a reference line. (**E**,**F**) KEGG enrichment analysis of DEGs. The red boxes highlight the key pathways of interest discussed in this study. (**G**–**I**) Heatmaps of DEGs enriched in key KEGG pathways, including (**G**) peroxisome and glutathione metabolism, (**H**) starch and sucrose metabolism, and (**I**) terpenoid backbone biosynthesis. Adjacent dots indicate statistical significance (*padj* < 0.05) for pairwise comparisons. (**J**–**L**) Functional mapping of key DEGs onto their corresponding metabolic pathways, including (**J**) Integrated antioxidant network, (**K**) Starch and sucrose metabolism pathway, and (**L**) Terpenoid backbone biosynthesis pathway. Solid arrows indicate enzymatic reactions; dashed arrows denote steps not directly measured in this study.

Both principal component analysis (PCA) and hierarchical clustering revealed close clustering of biological replicates within each treatment group and clear separation of transcriptional profiles among treatments (Figure 2A,B). Compared with the Al_0_ group, 642 DEGs (343 upregulated, 299 downregulated) were identified in the Al_1_ group, whereas 3019 DEGs (1415 upregulated, 1604 downregulated) were identified in the Al_10_ group (Figure 2C,D). Furthermore, KEGG enrichment analysis revealed distinct pathway responses. In the Al_1_ vs. Al_0_ comparison, DEGs were mainly enriched in Peroxisome, Glutathione metabolism, and Starch and sucrose metabolism pathways. In the Al_10_ vs. Al_0_ comparison, these pathways remained enriched, and Terpenoid backbone biosynthesis was additionally identified as a top-ranked pathway (Figure 2E,F). The enriched pathways were biologically relevant to the observed stress responses. The enrichment of peroxisome- and glutathione-related pathways was consistent with changes in ROS metabolism and cellular redox buffering. Alterations in starch and sucrose metabolism suggested changes in carbon utilization under Al^3+^ exposure, whereas enrichment of terpenoid backbone biosynthesis suggested transcriptional regulation of a precursor-supplying pathway associated with triterpenoid formation. Collectively, these pathway-level patterns suggested an association among redox regulation, carbohydrate metabolism, and secondary metabolite accumulation. Finally, qRT-PCR analysis of nine representative DEGs from these pathways showed expression patterns consistent with the RNA-seq results (Figure S3), further supporting the reliability of the transcriptomic analysis.

### 3.3. Transcriptional Regulation of DEGs Involved in Antioxidant Defense, Carbon Metabolism, and Terpenoid Biosynthesis

Based on the KEGG enrichment results, genes associated with antioxidant defense, carbohydrate metabolism, and terpenoid biosynthesis were further analyzed (Table 1). A subset of the pathway-associated genes was annotated as hypothetical proteins in the NR database; KEGG orthology assignments therefore provided complementary functional context. Core antioxidant enzymes in the Peroxisome and Glutathione metabolism pathways, including *GR*, *SOD*, *POD*, and *CAT*, were significantly upregulated under both Al^3+^ concentrations, indicating enhanced capacity for ROS detoxification. In contrast, *GPx* showed a modest but non-significant upregulation at 1 mM Al^3+^, whereas it was markedly upregulated at 10 mM (Figure 2G,J). Al^3+^ treatment also affected the expression of genes related to carbon metabolism (Figure 2H,K). Genes involved in intracellular sugar utilization, including *BGL* and *MGAM*, were significantly upregulated in a concentration-dependent manner under Al^3+^ treatment. By contrast, genes encoding extracellular cellulolytic enzymes, including *EG* and *CBH*, were upregulated (*EG2* significantly; *EG1* and *CBH* non-significantly) at 1 mM Al^3+^ but were markedly repressed at 10 mM. This pattern suggests a shift from extracellular degradation to intracellular mobilization of carbohydrate reserves under high Al^3+^ stress. Finally, genes involved in Terpenoid backbone biosynthesis also showed concentration-dependent responses (Figure 2I,L). *AACT* was significantly upregulated under both Al^3+^ treatments. *HMGS* and *HMGR* showed significant downregulation under 1 mM Al^3+^, whereas both genes were significantly upregulated at 10 mM, although the increase in *HMGR* expression was modest in magnitude. This pattern suggests that different Al^3+^ concentrations can lead to specific regulation of triterpenoid biosynthesis genes. Collectively, these results indicate that Al^3+^ induces a coordinated response involving multiple metabolic pathways.

### 3.4. Detection of Al^3+^-Induced Changes in Oxidative Stress Markers and Antioxidant Defense Systems

**Figure 3:**
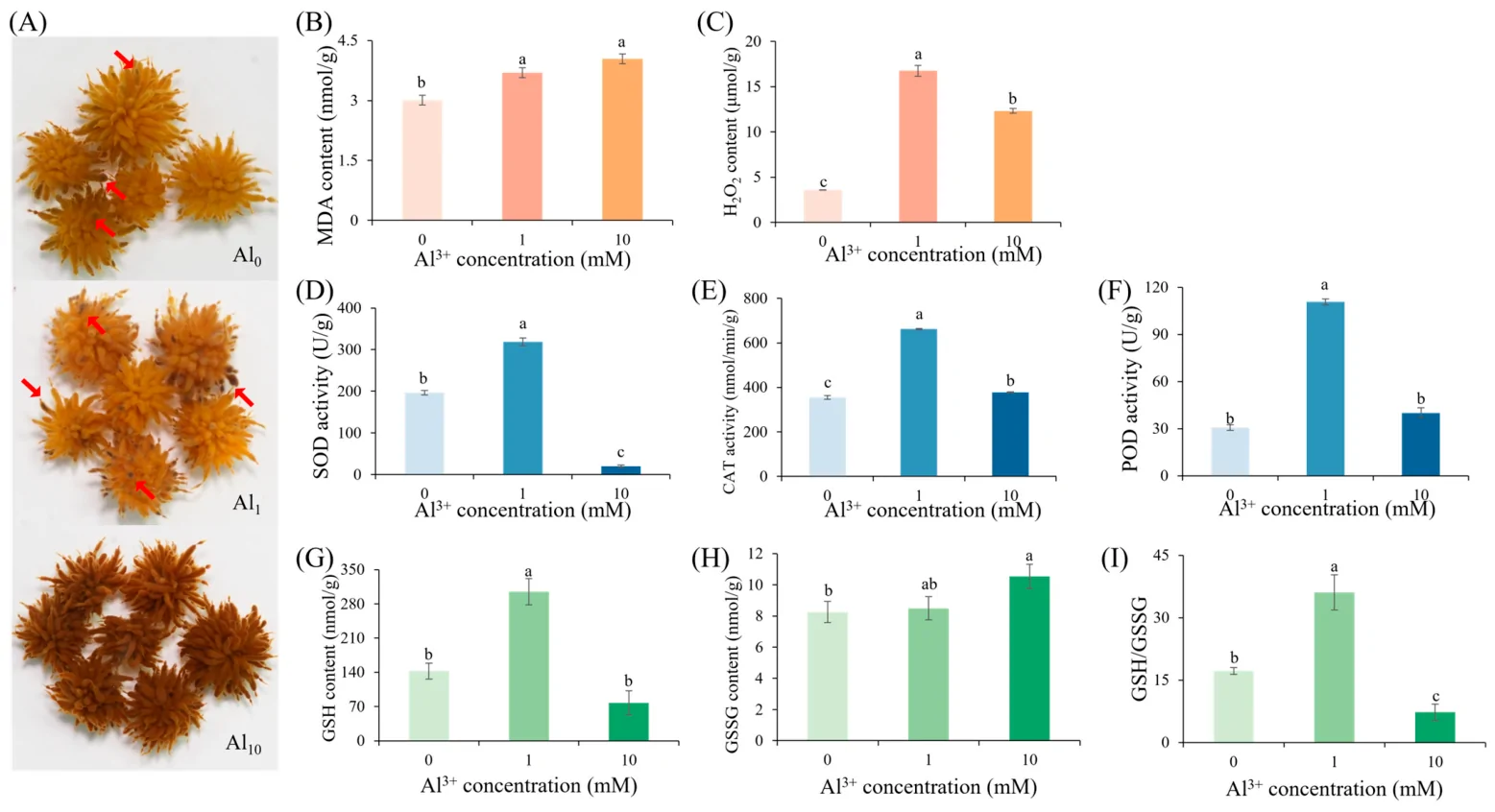
Oxidative stress markers and antioxidant defense systems in *S. baumii* mycelia exposed to varying Al^3+^ concentrations. (**A**) NBT staining of mycelia after 48 h of treatment. Red arrows indicate regions with formazan precipitation. (**B**) MDA content. (**C**) H_2_O_2_ content. (**D**) SOD activity. (**E**) CAT activity. (**F**) POD activity. (**G**) GSH content. (**H**) GSSG content. (**I**) GSH/GSSG ratio. Letters indicate a significant difference (*p* < 0.05).

To relate the transcriptomic changes to physiological responses, oxidative damage and ROS accumulation were evaluated following Al^3+^ exposure. Physiological parameters were normalized to mycelial fresh weight (FW). Absolute values and units are provided in Table S4. Biochemical measurements revealed that Al^3+^ treatment induced oxidative stress under both 1 mM and 10 mM conditions. MDA content was 1.23- and 1.34-fold higher than that of the control under 1 and 10 mM Al^3+^, respectively, while H_2_O_2_ content was 4.69- and 3.45-fold higher, respectively (Figure 3B,C). NBT staining further indicated that Al^3+^ concentration affected the accumulation pattern of O_2_^−^ in the mycelia (Figure 3A). In the control group, dark formazan precipitates were observed at the hyphal tips, reflecting basal metabolic activity. After exposure to 1 mM Al^3+^, this tip-localized staining became more intense and more widely distributed, indicating increased O_2_^−^ production. Under 10 mM Al^3+^, however, the mycelia displayed dark brown pigmentation even before staining, and no discernible color change was detected after NBT incubation. Because endogenous pigmentation could obscure the visual detection of NBT-derived formazan, particularly under the 10 mM treatment, the NBT images were interpreted only qualitatively and were not used to estimate the magnitude of oxidative stress. Instead, the increased MDA and H_2_O_2_ contents, together with the decreased GSH/GSSG ratio, provided quantitative evidence of redox disturbance.

To further assess how *S. baumii* responded to Al^3+^-induced oxidative stress, both enzymatic and non-enzymatic antioxidant markers were measured (Figure 3D–I). Under 1 mM Al^3+^, the antioxidant enzyme activities increased substantially. SOD, CAT, and POD activities were 1.62-, 1.87-, and 3.61-fold that of the control, respectively. Concurrently, GSH content was 2.14-fold that of the control, whereas GSSG content remained stable, resulting in a significantly higher GSH/GSSG ratio. This coordinated upregulation suggests an effective mechanism for maintaining redox homeostasis. In contrast, the 10 mM Al^3+^ treatment was associated with marked impairment of antioxidant capacity. SOD activity decreased to 10.06% of that of the control, and GSH content declined to 54.69% of that of the control, accompanied by a significant accumulation of GSSG and a sharp decline in the GSH/GSSG ratio. Together, these results indicate that the two Al^3+^ concentrations exerted profoundly different effects on cellular redox status. The 1 mM treatment activated antioxidant defenses and maintained redox homeostasis, whereas the 10 mM treatment disrupted redox balance and caused more pronounced oxidative damage.

### 3.5. Al^3+^-Induced Accumulation of Soluble Sugars and Total Triterpenoids in S. baumii

**Figure 4:**
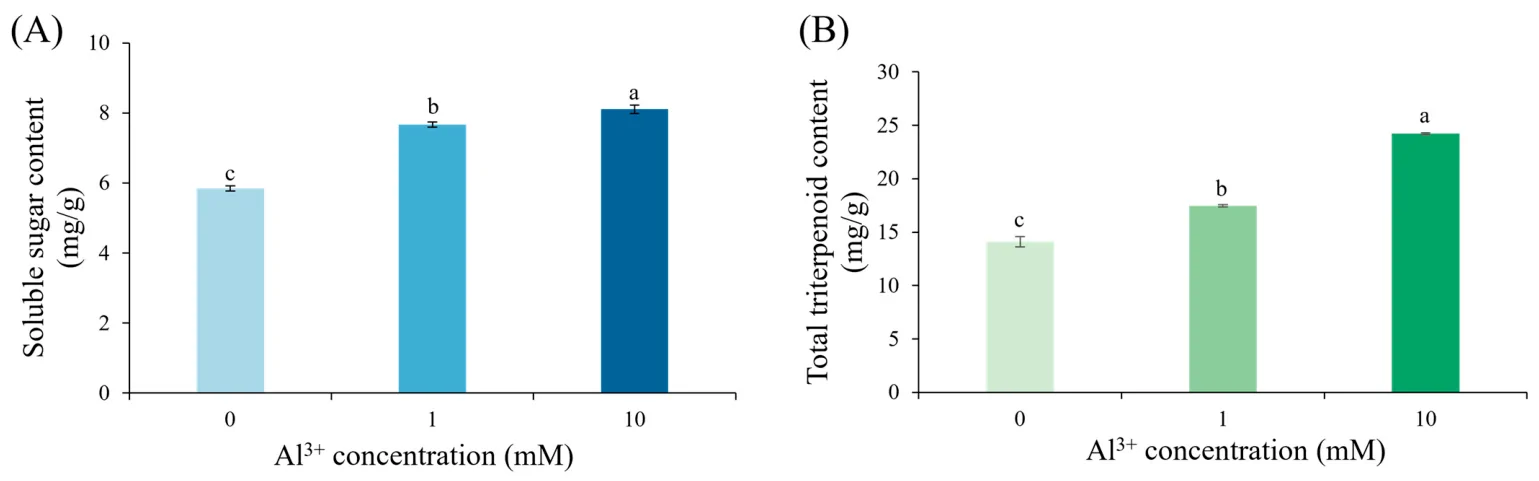
Detection of soluble sugar content and total triterpenoid content in *S. baumii* under Al^3+^ treatment. (**A**) Soluble sugar content. (**B**) Total triterpenoid content. Letters indicate a significant difference (*p* < 0.05).

Soluble sugar and total triterpenoid contents were measured to assess metabolic changes following Al^3+^ treatment (Figure 4). Both metabolites increased in a concentration-dependent manner. Soluble sugar content was 5.84 ± 0.08 mg/g in the control, 7.67 ± 0.08 mg/g under 1 mM Al^3+^, and 8.10 ± 0.12 mg/g under 10 mM Al^3+^, representing 1.31- and 1.39-fold that of the control, respectively. Total triterpenoid content was 14.11 ± 0.47 mg/g in the control, 17.46 ± 0.11 mg/g under 1 mM Al^3+^, and 24.22 ± 0.10 mg/g under 10 mM Al^3+^, corresponding to 1.24- and 1.72-fold that of the control, respectively. These results are consistent with the transcriptional upregulation of terpenoid backbone biosynthesis genes observed under 10 mM Al^3+^ stress and suggest that Al^3+^-induced metabolic reprogramming promotes the accumulation of both carbon reserves and bioactive secondary metabolites.

## 4. Discussion

Al^3+^ is a common environmental factor that can exert concentration-dependent effects on cellular physiology. In the present study, we investigated the dose-dependent effects of Al^3+^ on mycelial growth and metabolism in *S. baumii* by integrating transcriptomic, physiological, and biochemical analyses.

At the lower Al^3+^ concentration (1 mM), the antioxidant system remained functional and responsive. The coordinated upregulation of *SOD*, *CAT,* and *POD* transcripts was consistent with the increased activities of the corresponding enzymes, indicating activation of the antioxidant enzyme system. In parallel, *GR* was induced, whereas *GPx* showed relatively limited transcriptional change, and the GSH/GSSG ratio remained high. This pattern suggests that GSH was maintained at a high level and served as a redox buffer rather than being consumed in *GPx*-mediated H_2_O_2_ scavenging. Under the 1 mM treatment, the concurrent increases in H_2_O_2_ and MDA contents and mycelial growth were consistent with, but did not demonstrate, a possible role for moderate redox changes in growth-related signaling [18,19,20]. Establishing such a causal relationship will require direct redox perturbation experiments.

In stark contrast, exposure to 10 mM Al^3+^ produced a markedly different pattern. Although antioxidant genes remained transcriptionally induced, SOD activity decreased to merely 10.06% of the control, whereas CAT and POD activities declined relative to 1 mM treatment but remained near or above control levels. These results indicate that high Al^3+^ exposure caused dysregulation of the antioxidant system. The discordance between transcript abundance and enzyme activity may reflect post-translational regulation, altered protein abundance, or direct or indirect enzyme inactivation under high Al^3+^ exposure [21,22,23]. However, these mechanisms were not examined in the present study. Distinguishing among these possibilities will require proteomic analyses and direct enzyme-inhibition assays under defined Al^3+^ conditions. The antioxidant enzyme system served as the first line of defense against oxidative stress. The cell then engaged the glutathione system as a secondary defense, consuming GSH and leading to GSSG accumulation and a substantially reduced GSH/GSSG ratio. However, this compensatory effort proved insufficient, and redox balance was disrupted, resulting in pronounced oxidative damage and growth inhibition. Notably, H_2_O_2_ content was lower under the 10 mM treatment than under the 1 mM treatment, whereas MDA content continued to increase. The lower steady-state H_2_O_2_ concentration does not necessarily indicate weaker oxidative stress, because H_2_O_2_ content reflects the balance between production and removal, whereas MDA is an indicator of accumulated membrane lipid peroxidation. The pronounced decrease in SOD activity at 10 mM may have altered the conversion of superoxide to H_2_O_2_. However, this interpretation remains hypothetical because individual ROS species, ROS production pathways, and ROS removal fluxes were not directly quantified. Contributions from other ROS, impaired redox buffering, and treatment-dependent temporal dynamics cannot be excluded. This redox imbalance was accompanied by mycelial darkening in liquid culture and progressive intensification of colony pigmentation on solid medium, whereas the yellow color had faded under 1 mM Al^3+^ when redox balance was maintained. The intensified yellow-brown pigmentation observed under higher aluminum exposure may reflect the accumulation or oxidation of stress-associated pigments, phenolic compounds, or other secondary metabolites [24]. However, because no pigment-specific chemical analysis was performed, the pigment cannot be assigned to melanin or any other defined compound. Its chemical composition and biosynthetic origin therefore require targeted investigation.

The redox-associated changes described above were further reflected in carbon metabolism. Indeed, soluble sugars serve as key stress-responsive metabolites, and their dynamic changes are widely recognized as sensitive indicators of cellular metabolic status and adaptive strategies under various abiotic stresses [25,26]. Under 1 mM Al^3+^, the increased soluble sugar likely supported biomass production by providing carbon skeletons and energy. Under 10 mM Al^3+^, accumulated soluble sugars may contribute to osmotic stabilization and could potentially provide reducing power for the NADPH-dependent antioxidant system, thereby indirectly helping to mitigate oxidative stress [27,28].

The response of triterpenoid metabolism is of particular interest because triterpenoids are among the major bioactive constituents of *Sanghuangporus* spp. Under the 10 mM treatment, several genes associated with terpenoid backbone biosynthesis were upregulated, accompanied by an increase in total triterpenoid content. Similar stress-associated stimulation of triterpenoid accumulation has been reported in *Ganoderma lucidum* [18,29]. Notably, despite severe oxidative stress, triterpenoids continued to accumulate significantly. This uncoupling of secondary metabolism from primary growth suggests a resource reallocation from growth to chemical defense. Given the demonstrated antioxidant activity of *Sanghuangporus* triterpenoids [30,31], this accumulation may represent a metabolite-based defense strategy deployed when both enzymatic and non-enzymatic antioxidant defenses have been overwhelmed. From a bioprocess perspective, an increase in product content per unit biomass does not necessarily translate into greater volumetric productivity. Based on the mean biomass and total triterpenoid contents, the estimated volumetric yields were 56.86, 88.00, and 60.31 mg/L under the 0, 1, and 10 mM treatments, respectively. Among the tested conditions, the 1 mM treatment therefore produced the highest volumetric output, whereas the increased triterpenoid content at 10 mM was offset by the reduction in biomass. Future process development studies should evaluate a broader aluminum concentration range and optimize exposure timing and nutrient conditions. Residual aluminum content, product safety, downstream processing, and batch-level productivity must also be assessed before aluminum-based elicitation can be considered for practical application. In addition, genes associated with terpenoid backbone biosynthesis may provide candidate targets for future functional validation and metabolic engineering.

Collectively, these findings suggest that redox homeostasis may act as a central hub linking metal stress to metabolic adaptation in *S. baumii*, driving a trade-off between growth and defense. This is consistent with broader evidence that ROS signaling and redox regulation play key roles in stress adaptation and development in other macrofungi such as *Hypsizygus marmoreus*, *Ganoderma lucidum*, and *Ophiocordyceps sinensis* [32,33,34].

This study characterized the response of *S. baumii* at a single endpoint, 48 h after aluminum exposure, and therefore did not resolve the temporal sequence of early and late responses. Moreover, the exposure duration was selected on the basis of preliminary experiments involving other metal ions rather than an aluminum-specific time-course experiment. Future studies incorporating multiple sampling points will be required to distinguish early signaling events from later metabolic and damage-associated responses. In addition, the solid- and liquid-culture experiments showed the same directional responses at 1 and 10 mM; however, they differed in exposure timing, mycelial growth stage, physical state of the medium, and culture conditions. Consequently, the absolute effect sizes obtained from the two systems should not be compared directly. Finally, antioxidant enzyme activities were normalized to mycelial fresh weight rather than to total protein content. Direct comparisons with studies reporting activities in units per milligram of protein should therefore be made cautiously.

## 5. Conclusions

In conclusion, this study demonstrates that Al^3+^ uptake induces a concentration-dependent redox shift in *S. baumii*, which appears to influence the trade-off between primary growth and secondary metabolism. At 1 mM, functional enzymatic and non-enzymatic antioxidant defenses help preserve redox homeostasis, correlating with enhanced mycelial growth and soluble sugar accumulation. At 10 mM, the impairment of these defenses is associated with oxidative injury, growth inhibition, and a marked increase in total triterpenoid content. These findings suggest that redox status may act as a central hub linking metal stress to metabolic adaptation in this fungus, and provide a potential theoretical basis for the use of metal elicitors to modulate the production of bioactive compounds in medicinal macrofungi.

## Figures and Tables

**Table 1:** DEGs related to antioxidant defense and metabolism in *S. baumii* transcriptome.

KEGG Pathway	Gene ID	Gene Name	NR Annotation	KEGG	log2 (FoldChange)	
					Al_1_/Al_0_	Al_10_/Al_0_
Peroxisome and Glutathione metabolism	OCB90064.1	*GPx*	hypothetical protein A7U60_g2728	K00432	0.43	3.50 *
	OCB86551.1	*GR*	glutathione reductase	K00383	0.41 *	2.01 *
	OCB88914.1	*SOD*	Cu-Zn superoxide dismutase	K04565	1.25 *	2.69 *
	OCB85293.1	*POD*	Dyp-type peroxidase	-	0.50 *	4.17 *
	OCB92026.1	*CAT*	catalase	K03781	1.96 *	2.64 *
Starch and sucrose metabolism	OCB89527.1	*MGAM*	hypothetical protein A7U60_g3322	K01187	1.78 *	5.95 *
	OCB88885.1	*BGL1*	beta-glucosidase	K05349	0.94 *	1.97 *
	OCB84898.1	*BGL2*	glycoside hydrolase family 1 protein	K01188	0.52 *	4.20 *
	OCB90205.1	*EG2*	Cellulase	K01179	1.27 *	−3.04 *
	OCB87854.1	*EG1*	hypothetical protein A7U60_g4988	K01179	0.04	−1.74 *
	OCB90904.1	*CBH*	cellobiohydrolase	K01225	0.09	−1.38 *
Terpenoid backbone biosynthesis	QHI34614.1	*AACT*	acetyl-CoA acetyltransferase	K00626	0.80 *	1.66 *
	OCB86311.1	*HMGS*	hydroxymethylglutaryl-CoA synthase	K01641	−0.80 *	2.96 *
	QIJ32327.1 3	*HMGR*	3-hydroxy-3-methylglutaryl-coenzyme A reductase	K00021	−0.65 *	1.14 *

Note**:** *, *padj* < 0.05.

## Data Availability

All data directly supporting the conclusions of this manuscript are provided in the main text and Supplementary Materials. Additional data may be requested from the corresponding author upon reasonable request.

## Supplementary Materials

The following supporting information can be downloaded at https://www.mdpi.com/article/10.3390/jof12080586/s1: Figure S1: Al^3+^ content of *S. baumii* mycelia under different treatments; Figure S2: Length distribution of assembled unigenes; Figure S3: Comparison of gene expression levels from qRT-PCR and transcriptomic data; Table S1: Primers used for qRT-PCR; Table S2: Summary statistics for the de novo transcriptome assembly; Table S3: RNA-seq data statistics of *S. baumii*; Table S4: Absolute values of antioxidant enzyme activities and glutathione-related parameters in *S. baumii* mycelia under different Al^3+^ treatments.

## References

[1] Liu M.M., Zeng P., Li X.T., Shi L.G. (2016). Antitumor and immunomodulation activities of polysaccharide from *Phellinus baumii*. Int. J. Biol. Macromol..

[2] Yang Y., He P., Li N. (2019). The antitumor potential of extract of the oak bracket medicinal mushroom *Inonotus baumii* in SMMC-7721 tumor cells. Evid.-Based Complement. Altern. Med..

[3] Yang K., Zhang S., Ying Y.M., Li Y.G., Cai M., Guan R.F., Hu J.R., Sun P.L. (2020). Cultivated fruit body of *Phellinus baumii*: A potentially sustainable antidiabetic resource. ACS Omega.

[4] Huang G.Z., Zang F., Zhao C.Y., Wang H., Xi Y.L. (2024). Growth simulation of *Lyophyllum decastes* and *Coprinus comatus* and their influencing factors in a forested catchment. Forests.

[5] Ren A., Shi L., Zhu J., Yu H.S., Jiang A.L., Zheng H.H., Zhao M.W. (2019). Shedding light on the mechanisms underlying the environmental regulation of secondary metabolite ganoderic acid in *Ganoderma lucidum* using physiological and genetic methods. Fungal Genet. Biol..

[6] Sakamoto Y. (2018). Influences of environmental factors on fruiting body induction, development and maturation in mushroom-forming fungi. Fungal Biol. Rev..

[7] Long L., Jia M.H., Feng S., Long Z.F., Xu H. (2025). UIon antagonism strategy for cadmium mitigation in *Morchella sextelata*: Physiological and metabolomic insights. Fungal Biol..

[8] Zhang B., Zhou J., Li Q., Gan B., Peng W.H., Zhang X.P., Tan W., Jiang L.H., Li X.L. (2019). Manganese affects the growth and metabolism of *Ganoderma lucidum* based on LC-MS analysis. PeerJ.

[9] Kidd P., Proctor J. (2000). Effects of aluminium on the growth and mineral composition of *Betula pendula* Roth. J. Exp. Bot..

[10] Panda S.K., Matsumoto H. (2010). Changes in antioxidant gene expression and induction of oxidative stress in pea (*Pisum sativum* L.) under Al stress. Biometals.

[11] Yu Y., Zhou W.W., Zhou K.J., Liu W.J., Liang X., Chen Y., Sun D.S., Lin X.Y. (2018). Polyamines modulate aluminum-induced oxidative stress differently by inducing or reducing H_2_O_2_ production in wheat. Chemosphere.

[12] Liu L.Y., Sakai K., Tanaka T., Kusumoto K.I. (2024). Morphological responses of two *Aspergillus oryzae* strains to various metal ions at different concentrations. Mycoscience.

[13] Chen R.R., Zhu Q., Fang Z.J., Huang Z.W., Sun J., Peng M., Shi P. (2020). Aluminum induces oxidative damage in *Saccharomyces cerevisiae*. Can. J. Microbiol..

[14] Lv Y.R., Li A., Gu X.R., Hu J., Wen S.J., Xu S.R., Deng Y.X., Chen D.M., He X.H. (2026). Integrative omics analyses of ectomycorrhizal fungi modulate root growth and defense for mitigating aluminum toxicity in *Pinus massoniana*. Tree Physiol..

[15] Gu X.R., Jia H., Wang X.H., Jiang Y.N., Li J., He X.H. (2023). Differential aluminum tolerance and absorption characteristics in *Pinus massoniana* seedlings colonized with ectomycorrhizal fungi of *Lactarius deliciosus* and *Pisolithus tinctorius*. J. For. Res..

[16] Liu Z.C., Si C.Y., Xiao W.Q., Wang A.X., Zou L. (2025). Identification of a key gene involved in triterpenoid biosynthesis in *Sanghuangporus baumii* under Mn^2+^ induction and its regulatory role. BMC Plant Biol..

[17] Ghuniem M.M. (2024). Determination of some element’s migrants in aqueous simulant from plastic food contact products by inductively coupled plasma mass spectrometer. Food Anal. Methods.

[18] Gao T., Shi L., Zhang T.J., Ren A., Jiang A.L., Yu H.S., Zhao M.W. (2018). Cross Talk between calcium and reactive oxygen species regulates hyphal branching and ganoderic acid biosynthesis in *Ganoderma lucidum* under copper stress. Appl. Environ. Microbiol..

[19] Hong Y.H., Boiti A., Vallone D., Foulkes N.S. (2024). Reactive oxygen species signaling and oxidative stress: Transcriptional regulation and evolution. Antioxidants.

[20] Mu D.S., Li C.Y., Zhang X.C., Li X.B., Shi L., Ren A., Zhao M.W. (2014). Functions of the nicotinamide adenine dinucleotide phosphate oxidase family in *Ganoderma lucidum*: An essential role in ganoderic acid biosynthesis regulation, hyphal branching, fruiting body development, and oxidative-stress resistance. Environ. Microbiol..

[21] Feng M., Yin H., Peng H., Liu Z.H., Lu G.N., Dang Z. (2017). Hexavalent chromium induced oxidative stress and apoptosis in *Pycnoporus sanguineus*. Environ. Pollut..

[22] Krumova E., Kostadinova N., Miteva-Staleva J., Gryshko V., Angelova M. (2016). Cellular response to Cu- and Zn-induced oxidative stress in *Aspergillus fumigatus* isolated from polluted soils in Bulgaria. Clean–Soil Air Water.

[23] Zhang Q.H., Zeng G.M., Chen G.Q., Yan M., Chen A.W., Du J.J., Huang J., Yi B., Zhou Y., He X.X. (2015). The effect of heavy metal-induced oxidative stress on the enzymes in white rot fungus *Phanerochaete chrysosporium*. Appl. Biochem. Biotechnol..

[24] Tong C.Q., Luo J., Xie C.L., Wei J.H., Pan G.Q., Zhou Z.Y., Li C.F. (2023). Characterization and biological activities of melanin from the medicinal fungi *Ophiocordyceps sinensis*. Int. J. Mol. Sci..

[25] Qi W.J., Bai J.P., Yu H., Han G.J. (2025). Physiological adaptations of *Vigna radiata* to heavy metal stress: Soluble sugar accumulation and biomass enhancement. Plants.

[26] Cui Y.N., Yan S.J., Zhang Y.N., Wang R., Song L.L., Ma Y., Guo H., Yang P.Z. (2024). Physiological, metabolome and gene expression analyses reveal the accumulation and biosynthesis pathways of soluble sugars and amino acids in sweet sorghum under osmotic stresses. Int. J. Mol. Sci..

[27] Rosa M., Prado C., Podazza G., Interdonato R., González J.A., Hilal M., Prado F.E. (2009). Soluble sugars: Metabolism, sensing and abiotic stress: A complex network in the life of plants. Plant Signal. Behav..

[28] Couée I., Sulmon C., Gouesbet G., Amrani A.E. (2006). Involvement of soluble sugars in reactive oxygen species balance and responses to oxidative stress in plants. J. Exp. Bot..

[29] Xu N.Y., Xiao X.X., Zhong J.J. (2014). Induction of ganoderic acid biosynthesis by Mn^2+^ in static liquid cultivation of *Ganoderma lucidum*. Biotechnol. Bioeng..

[30] Cai C.S., Ma J.X., Han C.R., Jin Y., Zhao G.Z., He X.W. (2019). Extraction and antioxidant activity of total triterpenoids in the mycelium of a medicinal fungus, *Sanghuangporus sanghuang*. Sci. Rep..

[31] Liu Z.C., Liu R.P., Zou L. (2023). Development of a transformation system for the medicinal fungus *Sanghuangporus baumii* and acquisition of high-value strain. Mycobiology.

[32] Tong X.X., Wang F., Zhang H., Bai J., Dong Q., Yue P., Jiang X.Y., Li X.R., Wang L., Guo J.L. (2021). iTRAQ-based comparative proteome analyses of different growth stages revealing the regulatory role of reactive oxygen species in the fruiting body development of *Ophiocordyceps sinensis*. PeerJ.

[33] You B.J., Chang W.T., Chung K.R., Kuo Y.H., Yang C.S., Tien N., Hsieh H.C., Lai C.C., Lee H.Z. (2012). Effect of solid-medium coupled with reactive oxygen species on ganoderic acid biosynthesis and MAP kinase phosphorylation in *Ganoderma lucidum*. Food Res. Int..

[34] Zhang J.J., Hao H.B., Wu X.L., Wang Q., Chen M.J., Feng Z.Y., Chen H. (2020). The functions of glutathione peroxidase in ROS homeostasis and fruiting body development in *Hypsizygus marmoreus*. Appl. Microbiol. Biotechnol..

