# Supplementary material for: Transcriptomics and Physiological Analysis Reveal Response Strategies of Sanghuangporus baumii to Aluminum Exposure
Source: J Fungi (Basel). 2026 Aug 7;12(8):586. doi: 10.3390/jof12080586 (PMC13514426; doi:10.3390/jof12080586)
Supplement: Supplementary file 1 [file jof-12-00586-s001.zip › jof-4453756-supplementary.pdf]

Transcriptomics and Physiological Analysis Reveal Response Strategies of *Sanghuangporus baumii* to Aluminum Exposure

Xinyu Tong, Anxin Wang, Zengcai Liu * and Li Zou *

College of Forestry, Northeast Forestry University, Harbin 150040, China; 18846783859@163.com (X.T.); 17661291539@163.com (A.W.)

* Correspondence: 1758458181@nefu.edu.cn (Z.L.); shyj@nefu.comedu.cn (L.Z.)

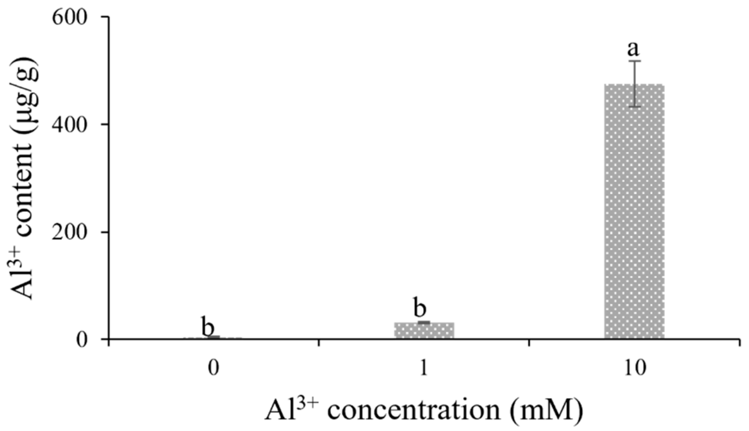

Figure S1. Al^{3+} content of *S. baumii* mycelia under different treatments.

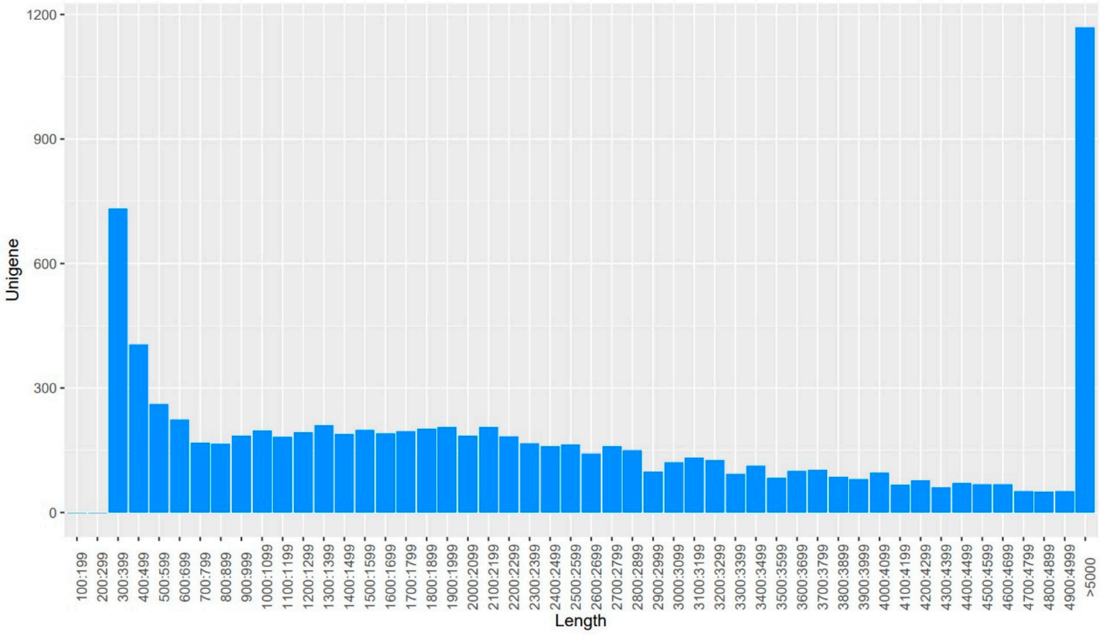

Figure S2. Length distribution of assembled unigenes.

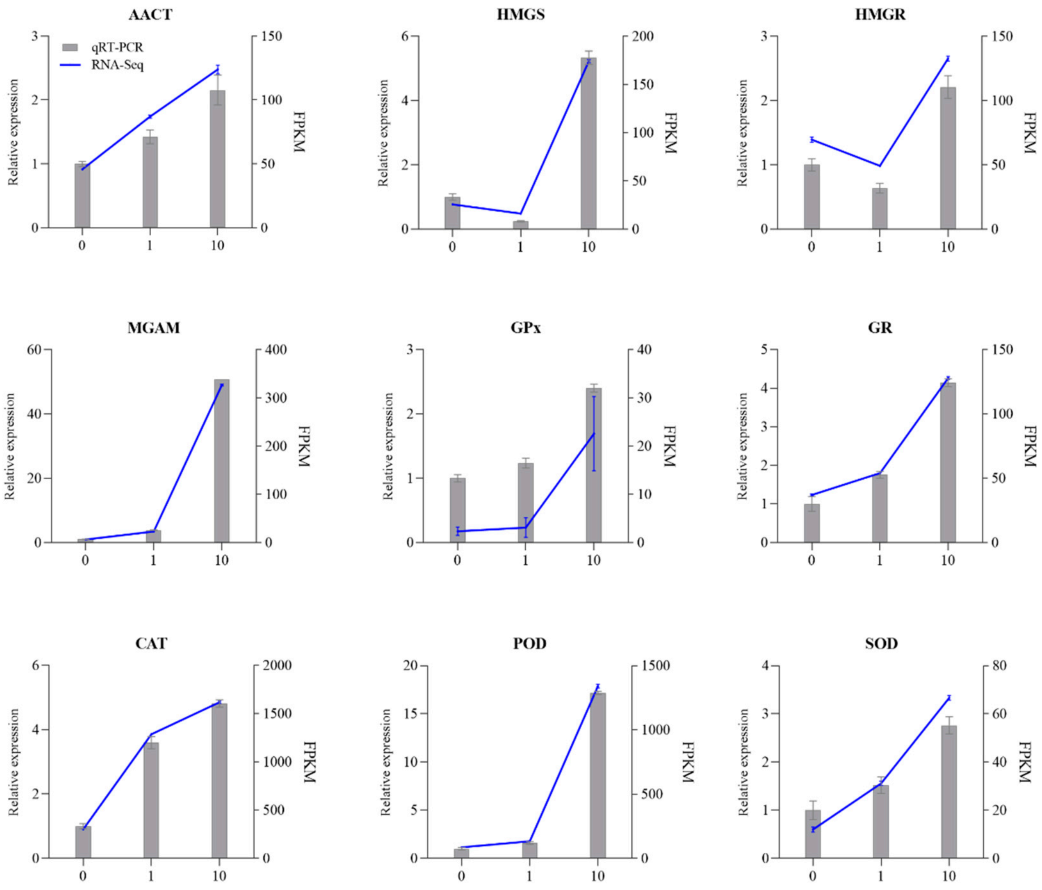

Figure S3. Comparison of gene expression levels from qRT-PCR and transcriptomic data.
